# Linc00514 promotes breast cancer metastasis and M2 polarization of tumor-associated macrophages via Jagged1-mediated notch signaling pathway

**DOI:** 10.1186/s13046-020-01676-x

**Published:** 2020-09-17

**Authors:** Sifeng Tao, Qiang Chen, Chen Lin, Haiying Dong

**Affiliations:** 1 Department of Breast Surgery, The Second Affiliated Hospital, College of Medicine, Zhejiang University, 88 Jiefang Rd, Hangzhou, 310009 People’s Republic of China; 2 Department of Urology, Zhejiang Provincial People’s Hospital, Hangzhou Medical College, Hangzhou, 310009 China

**Keywords:** linc00514, Jagged1, Breast cancer, Tumor-associated macrophage

## Abstract

**Background:**

Tumor-associated macrophages (TAMs) and tumor cells are important components of the tumor microenvironment. M2 polarization of TAMs, which is a major actor in breast cancer malignancy and metastasis, can be induced by breast cancer cells. However, the potential mechanisms of the interaction between breast cancer cells and TAMs remain unclear.

**Methods:**

The candidate breast cancer-associated long non-coding RNAs (lncRNAs) were analyzed using the GEO database. Functional assays, including MTT assay, Transwell assay, and EdU labeling detection, were performed to investigate the oncogenic role of linc00514 in breast cancer progression. The co-culture and ELISA assays were used to assess the role of linc00514 in macrophage recruitment and M2 polarization. RNA immunoprecipitation, RNA pull-down, and luciferase reporter assays were applied to determine the mechanism of linc00514 in breast cancer metastasis. Mouse xenograft models, mouse pulmonary metastatic models, and mouse primary tumor models were used to assess the role of linc00514 in M2 macrophage polarization and breast cancer tumorigenicity.

**Results:**

Linc00514 was highly expressed in clinical breast cancer tissues and breast cancer cell lines. Overexpression of linc00514 promoted the proliferation and invasion of breast cancer cells and increased xenograft tumor volumes and pulmonary metastatic nodules. Overexpression of linc00514 also increased the percentage of macrophages expressing M2 markers CD206 and CD163. Mechanistically, linc00514 promoted Jagged1 expression in a transcriptional manner by increasing the phosphorylation of a transcription factor STAT3. Subsequently, Jagged1-mediated Notch signaling pathway promoted IL-4 and IL-6 secretions in breast cancer cells and ultimately inducing M2 polarization of macrophages.

**Conclusion:**

Linc00514 plays an important role in regulating breast cancer tumorigenicity and M2 macrophage polarization via Jagged1-mediated Notch signaling pathway.

## Background

Macrophages, as a type of immune cells ubiquitous in tissues and organs, are not only the innate immune cells to prevent infection, but also an important part of tumor growth and metastasis [1]. Tumor-associated macrophages (TAMs) are vital components in the tumor microenvironment (TME) [2]. Their functions are widely restricted and regulated by the other components in TME, such as tumor cells. By the interactions between TAMs and tumor cells, TAMs are widely involved in the tumorigenicity, including proliferation and invasion of tumor cells, promoting tumor growth and metastasis [3]. TAMs are derived from peripheral monocytes. The alternative polarization of macrophages, M1-like or M2-like, can be induced by different stimuli from tumor cells or other cells in TME [4]. Different from the anticancer effect of M1-like macrophages, M2-like macrophages secret oncogenic cytokines, growth factors, and proteases, thus increasing tumorigenicity [5]. Recent studies have revealed that the M2 polarization of TAMs can be induced by tumor cells in breast cancer. Weng et al. [6] found that the oncogene MCT-1 stimulated interleukin-6 (IL-6) secretion of triple-negative breast cancer cells that promoted M2 polarization of macrophages to enhance invasiveness. Ma et al. [7] found that the SIAH2-NRF1 Axis impairs the polarization of TAMs in breast cancer. Although the interplay between TAMs and tumor cells has been established, the mechanisms of tumor cells modulating TAM polarization remain unclear.

The Notch pathway cooperates in the initiation and progression of breast cancer metastasis. Lee et al. [8] found that the protein kinase Fyn plays a significant role in promoting breast cancer tumorigenicity via the Notch2 signaling pathway. Hu et al. [9] found that the application of Brucine could inhibit Notch1 pathway and thereby reduce bone metastasis of breast cancer. Jagged1, which is encoded by JAG1 gene, is a ligand of the Notch 1/2 signaling pathway. Breast cancer cells with high JAG1 mRNA and protein levels are more aggressive [10]. A positive correlation between Jagged1 expression and M2-like macrophage infiltration in breast cancer tissues was found in a clinical study [11]. In addition, it has been found in colon cancer that the Notch signaling pathway in colon cancer cells can affect the polarization and recruitment of TAMs by secreting IL-4 [12]. However, whether Jagged1-mediated Notch signaling pathway in breast cancer cells is involved in the M2 polarization of macrophages is still unknown.

Long noncoding RNAs (lncRNAs) are a group of non-coding RNAs (ncRNAs) longer than 200 nucleotides. Various studies have found that lncRNA are involved in the development of breast cancer [13–15]. LncRNAs have multiple functions [16]. They could bind with some proteins to generate functional ribonucleic acid protein (RNP) [17] or bind with some DNAs or RNAs to form complexes, thereby regulating the epigenetic modification of genes [18]. Signal transducer and activator of transcription 3 (STAT3) is a critical transcription factor which has been reported to be bound by lncRNAs [19, 20]. The phosphorylated STAT3 (pSTAT3) is translocated to the nucleus and increases the transcription of oncogenic genes. More importantly, Zhao et al. [21] found that nuclear STAT3 directly activated the transcription of Jagged1 in mouse pregranulosa cells. Yang et al. [22] found that the blocking of the STAT3 signaling abrogated IL-6-induced Jagged1 expression in human gastric cancer cells, suggesting STAT3 may regulate Jagged1-mediated Notch signaling pathway. Based on these findings, we assumed that lncRNAs in breast cancer cells may regulate the Notch signaling pathway via STAT3-mediated transcription modulation of Jagged1. Therefore, in the current study, we performed an integrated analysis of lncRNAs which are dysregulated in breast cancer cells, and found that linc00514/STAT3/Jagged1 axis is associated with the breast cancer tumorigenicity and macrophage M2 polarization.

## Materials and methods

### Clinical samples and cell lines

Breast cancer patients (*n* = 46) who were admitted to The Second Affiliated Hospital of Zhejiang University from January 2018 January of 2019 were enrolled. None of the patients received preoperative chemotherapy or radiotherapy. During the tumor resection operation, the fresh tumor tissues and the paired paracancer tissues were collected. All resected tissues were pathologically confirmed. Then the tissues were stored in a − 80 °C liquid nitrogen before further examinations. The basic characteristics of 46 patients are listed in Table 1.

**Table 1:** Correlation between linc00514 expression and clinicopathological characteristics of breast cancer patients

Parameters	linc00514 expression		*p*-value
	High (*n* = 23)	Low (*n* = 23)	
Age			
> =60	11	14	0.689
< 60	12	9	
Tumor Size			
T1 + T2	17	23	**0.028**
T3 + T4	6	0	
Lymph node involved			
No	10	14	0.237
Yes	13	9	
Metastasis to organs			
No	22	23	1
Yes	1	0	
Stage			
I + II	16	21	0.137
III + IV	7	2	
ER			
Negative	6	2	0.212
Positive	15	20	
PR			
Negative	7	2	0.114
Positive	14	20	
HER2			
Negative	1	2	0.946
Positive	13	11	

**Note:** Bold values indicate *P* < 0.05

**Abbreviation:** *ER* Estrogen receptor; *PR* Progesterone receptor

Human breast cancer cell lines, including MDA-MB-231 (triple negative), MDA-MB-468 (triple negative), and MCF-7 (ER positive), human normal breast epithelial cell line MCF-10A, human monocyte THP-1 cells, and mouse breast cancer cell line (4 T1), and HEK 293 T cells were obtained from American Type Culture Collection (ATCC; Manassas, VA, USA). MDA-MB-468 and MDA-MB-231 cells were cultured in the ATCC-formulated Leibovitz’s L-15 Medium (ATCC, Catalog No. 30–2008) supplemented with 10% fetal bovine serum (FBS; Gibco, Waltham, MA, USA). MCF-7 cells were cultured in the Dulbecco’s Minimum Essential Medium (DMEM; ATCC, Catalog No. 30–2002) supplemented with 10% FBS, 10 μg/mL human insulin (Sigma-Aldrich, St Louis, MO, USA), and 1 μM 4-hydroxytamoxifen. MCF-10A cells were cultured in the base medium obtained from Lonza/Clonetics Corporation as a kit supplemented with 100 ng/ml cholera toxin. THP-1 cells and 4 T1 cells were cultured in the RPMI-1640 medium (ATCC, Catalog No. 30–2001) supplemented with 10% FBS. HEK 293 T cells were cultured in the BalanCD HEK293 medium (Irvine Scientific, Catalog No. 91165) supplemented with 200 mM L-glutamine (ATCC, Catalog No. 30–2214) and ITS (Corning, Catalog No. 25–800-CR). All the cell lines were maintained in a container at 37 °C in 5% CO_2_.

### Quantitative real-time PCR

The expression of linc00514 in tissues and cells was detected using quantitative real-time PCR (qRT-PCR). The TRIzol regent was used to extract the total RNAs from tissues and cells. The PrimeScript RT reagent Kit (Takara, Tokyo, Japan) was used to reversely transcript RNAs to cDNAs. The spectrometer was used to detect the RNA concentrations. The SYBR Premix Ex Taq TM (Takara, Tokyo, Japan) was used to perform qRT-PCR procedures. GAPDH was used as the internal control. The relative gene expression was calculated using 2^−ΔCt^ or 2^-△△Ct^ method. The primers used in this study were shown in Table 2.

**Table 2:** The primers, small interfering RNA sequences, and probes used in this study

qRT-PCR Primers (from 5′ to 3′)	
Linc00514	F: GTGTGCCTGCCCTTTCCTGTG
	R: TCCCATCCCATTCCCATCACCAG
CD206	F: TTCAGTGGACCATCGAGGAAGAGG
	R: ATGGCAACACACCCTGGCTTTC
CD163	F: GCCACAACAGGTCGCTCATCC
	R: GCAAGCCGCTGTCTCTGTCTTC
Jag1	F: TGTGGCTTGGATCTGTTGCTTGG
	R: ACGTTGTTGGTGGTGTTGTCCTC
Hes1	F: GGCTGGAGAGGCGGCTAAGG
	R: TGCTGGTGTAGACGGGGATGAC
GAPDH	F: CAATGACCCCTTCATTGACC
	R: GACAAGCTTCCCGTTCTCAG
Si-RNA sequences	
Si-control	UUCUCCGAACGUGUCACGUCU
Si-linc00514	GGACCACACUGGGAUUCUAGA
Si-STAT3	GAGUCAAGGAGACAUGCAAGA
Si-jag1	GGGUCAGAAUUGUGACAUAAA
Si-jak2	GAUGGAUAUUAUAGAUUAACU
Agarose gel electrophoresis primers	
Linc00514	F: CAGGACCTTCGCAGGTGCTG
	R: CTTCTGGGGTTTTCCACCCCC
RNA Pull down Probe	
CATCCCTTCCTCTCTGCTCAGGACCAGGGCCAGGCCCCTACATCAGTAACCCCCAACCCCTAAGACCTCCATGCTCCCAGAACTCCAGGGCCTTACCTGCCTGGGTTCCAGCTCCCAGGAAGGCCCCGACTCCCCACCCATTCCTATTCCCAAATCCCAGGGGACAGAGCCAAAGAG	
FISH Probe	
GCCATGTGGAGTGCGGGTCCGTGGTCCAAACACCACCCAGGGCCACTGTGCTCCACACCCTGACAGTTCACTAAGGGCGCATGCCCAGGAAGCAGCTCAGAGGCCTCCACAGGAGTCCCCACCCACAGGAATGGCATTGGCAGGCCACAGAGGGACGAGTGCCTGGGGAGGGTAACACAGACCGAGAGGGGGACAGAGAGGGACAGGACATGGTGAGTGTGGCATGGAGGTGGCACACGGGCGGGGTCCTCCCTTCATCCGCTTCTGAGGTTCCCTCTTCCCCAGGCCTGGGGAGAGCTAGAAAGAGGCTGACATTGACCCTAAGAGCTCCTCCCCTAAGAATGAGGGTTGGGACCCTAAACCCTCCTGCCCGCCCCTCCCTGCGGCCCCTGCCCAGCCACCCTCACCTG	

Abbreviation: *si-RNA* Small interfering RNA; *FISH* Fluorescence in situ hybridization

### Cell viability and invasion

Cell viability of the breast cancer cell lines (MDA-MB-231, MDA-MB-468, MCF-7, and 4 T1) was detected using 3-(4,5-dimethylthiazol-2-yl)-2,5-diphenyltetrazolium bromide (MTT) assay [23] or 5-Ethynyl-2-deoxyuridine (EdU) labeling detection [24]. Briefly, cancer cells were cultured in 96-well plates (1 × 10^4^ cells/well) and were incubated for 24 h at 37 °C in 5% CO_2_. The MTT solution (100 μL) was added to each well, and the cells were incubated for 30 min at 37 °C. After the removal of the MTT solution, the plates were incubated for 15 min at 37 °C. The absorbance of each well was measured on an ELISA micro-plate reader (Bio-Rad, Hercules, CA, USA) at 450 nm. For EdU labeling detection, the breast cancer cells were seeded in a 96-well plate and exposed to 50 μM EdU (RiboBio, Guangzhou, China). Subsequently, we stained the DNA contents with Hoechst 33342 for 30 min and visualized them using a microscopy (Olympus, Tokyo, Japan).

Cell invasion capability of the breast cancer cell lines (MDA-MB-231, MDA-MB-468, and MCF-7) was detected using Transwell assay [25]. The upper chamber was coated with the Matrigel (Sigma, St. Louis, MO, USA). The breast cancer cells (1 × 10^5^ cells) were seeded in serum-free media in the upper chamber with noncoated membrane (8 μm in pore size; Millipore, Schaffhausen, Switzerland). Then the 20% FBS was added to the lower chamber. After 24 h, the migrated cells in the lower chamber were counted by using a microscope.

### Cell transfection

The knockdown and the overexpression of linc00514 in breast cancer cell lines (MDA-MB-231, MDA-MB-468, and MCF-7) were performed using the transfection of the small interfering RNAs (siRNAs) against linc00514 (Si-linc00514) and the linc00514-overexpressing plasmids (pcDNA-linc00514). The knockdown of STAT3 and JAG1 in breast cancer cell lines was performed using the transfection of siRNAs against STAT3 (Si-STAT3) and JAG1 (Si-JAG1). The Si-linc00514, Si-STAT3, and Si-JAG1 and their negative control (Si-control/Si-ctrl) as well as the pcDNA-linc00514 and its negative control (pcDNA) were obtained from RiboBio Corporation (Guangzhou, Guangdong, China). Cell transfection was performed using the Lipofectamine 2000 (Thermo Fisher Scientific, Waltham, MA, USA).

### Mouse xenograft model

Female Balb/c nude mice (5–7 weeks) were bought from Shanghai Lab Animal Research Center (Shanghai, China). Mice were randomly divided into 2 groups, the linc00514-OVE group (*n* = 5) and the control group (*n* = 5). Mice in the linc00514-OVE group were subcutaneously engrafted in the right-hind flank with MDA-MB-468 cells (5 × 10^6^ cells) which were transfected with pcDNA-linc00514 for 48 h. Mice in the control group were engrafted with the same volume of MDA-MB-468 cells which were transfected with pcDNA for 48 h. The tumors were measured on a weekly basis, and the tumor volume was ascertained as width^2^ × length × 0.5 (mm^3^). Seven weeks later, mice in the two groups were sacrificed and the tumor tissues were collected. Immunohistochemistry of the tumor tissues using the antibodies against Ki67, CD206, F4/80, pSTAT3, or Jagged 1 was performed.

### Mouse primary breast cancer model

Female Balb/c mice (6–8 weeks) were bought from Shanghai Lab Animal Research Center (Shanghai, China). They were randomly divided into 3 groups: the Si-ctrl group (*n* = 10), the Si-STAT3 group (*n* = 10), and the Si-JAG1 group (*n* = 10). After the transfection with Si-ctrl, Si-STAT3, or Si-JAG1 for 48 h, the mouse breast cancer cell line 4 T1 cells were suspended in 0.1 ml of serum-free DMEM. Then they were subcutaneously injected into the 4th coupled abdominal mammary glands of each mouse [26]. Each mammary gland was injected with 5 × 10^5^ 4 T1 cells. Twenty days later, mice were sacrificed and the tumor tissues as well as the lungs were collected. The tumor volumes were measured. Lungs were fixed in 10% formalin, and embedded in paraffin. Serial pathological sections from the embedded tissues were stained by the standard hematoxylin-eosin (HE) procedure to detect metastasis. Immunohistochemistry of the tumor tissues using the antibodies against CD206 and F4/80 was performed.

### In vivo metastatic experiments

Female Balb/c nude mice (5–7 weeks) were randomly divided into 2 groups, the linc00514-OVE group (*n* = 5) and the negative control (NC) group (*n* = 5). Mice in the linc00514-OVE group were intravenously injected with the MDA-MB-231 cell suspension [1× 10^6^ cells in 100 μL phosphate buffer saline (PBS)] which were transfected with pcDNA-linc00514 for 48 h. Mice in the NC group were intravenously injected with the same volume of the MDA-MB-231 cell suspension which were transfected with pcDNA for 48 h. The MCF-7 cell suspension (1.5 × 10^6^ cells in 100 μL PBS) was also used in the metastatic experiments. Six weeks later, lungs were collected and stained by the standard HE procedure to detect metastasis.

### Macrophage polarization experiments

The effect of breast cancer cells on the polarization of macrophages was detected using transwell method. The breast cancer cell lines (MDA-MB-231, MDA-MB-468, and MCF-7) which were transfected with Si-control, Si-linc00514, pcDNA, or pcDNA-linc00514 were added to the upper chamber. The upper chamber which was filled with the same volume of RPMI medium was used as the control group. THP-1 cells were added to the lower chamber. Before adding the breast cancer cells to the upper chamber, the THP-1 cells were treated with 100 nM phorbol-12-myristate-13-acetate (PMA) for 1 day to induce the polarization of the macrophages. After 48 h, the relative mRNA levels of CD206 and CD163, both of which are M2 macrophage markers, in the cells of the lower chamber were detected using qRT-PCR. The percentage of CD206/CD163 positive cells was detected using flow cytometry.

### Western blot analysis

The protein expressions of pSTAT3, STAT3, Jagged1 in breast cancer cell lines (MDA-MB-231, MDA-MB-468, 4 T1, and MCF-7) after cell transfection were detected using western blot analysis. The cell lysis buffer (Thermo Fisher Scientific, Waltham, MA, USA) was used to isolate proteins from cells. Bradford method was used to detect the protein concentration. After loading the proteins onto the 12% sodium dodecyl sulfate-polyacrylamide gel electrophoresis (SDS-PAGE), we transferred the proteins to PVDF membranes (Invitrogen, Waltham, MA, USA), blocked them with 5% skimmed milk, and incubated them with primary antibodies, including anti-pSTAT3 (ab76315, 1/2000; Abcam, Cambridge, UK), anti-STAT3 (ab68153, 1/2000; Abcam), anti-Jagged1 (ab109536, 1/1000; Abcam), anti-IL-4 (ab62351, 1/1000; Abcam), anti-IL-6 (ab6672, 1/500; Abcam), anti-JAK2 (ab108596, 1/5000; Abcam), and anti-β-actin (ab8226, 1/500; Abcam) at 4 °C overnight, followed by incubating them with horseradish peroxidase (HRP)-conjugated secondary antibody (goat anti-rabbit or goat anti-mouse; 1/3000; Cell Signaling Technology) for 1.5 h. Then the bands were visualized by the enhanced chemiluminescence kit (Thermo Scientific, Waltham, MA, USA) and the density of bands was analyzed using Image J software.

### Enzyme-linked immunosorbent assay

The cell-free supernatants from breast cancer cell lines (MDA-MB-231, MDA-MB-486 and MCF-7) were collected. The levels of IL-4, IL-6, IL-10, IL-13, and IL-35 in the supernatant of cell culture medium were detected using enzyme-linked immunosorbent assay (ELISA) according to the manufacturer’s instructions.

### Luciferase reporter assay

The human/mouse DNA sequences of the JAG1 promoter which are predicted to be bound with transcription factor STAT3 were cloned into a pGL3 vector. After the transfection with STAT3-overexpressing plasmids (STAT3 vector) or control empty plasmids (Empty vector), 293 T cells were incubated with reporter lysis buffer to obtain cell lysates, then the luciferase substrate was added. The dual-luciferase reporter system (Promega, Madison, WI, USA) was used to detect the luciferase activity to evaluate transcription activity of STAT3.

### Cell distribution of linc00514

The nuclear and cytoplasmic fractions of the breast cancer cell lines (MDA-MB-231, MDA-MB-468, and MCF-7) were isolated as previously described [27]. The expression of U6, GAPDH, and linc00514 in cytosol and nucleus was detected using qRT-PCR.

### Fluorescence in situ hybridization

The cellular localization of linc00514 was detected using fluorescence in situ hybridization (FISH) as previously described [28]. In brief, breast cancer cell lines (MDA-MB-231, MDA-MB-468, and MCF-7) were washed with PBS. Then they were fixed in 4% paraformaldehyde. After being treated with the protease reagent, the slides were incubated with prehybridization buffer for 4 h at 40 °C, followed by the hybridization with digoxin-labeled linc00514 probe overnight at 40 °C. Then the slides were incubated with biotin conjugated anti-digoxin antibody. The slides were then incubated with SABC-Cy3 at 37 °C for 30 min after washing, then they were visualized under a confocal microscope.

### RNA immunoprecipitation and RNA pull-down assay

The interaction between linc00514 and the JAK2/STAT3 compound was detected using RNA immunoprecipitation (RIP) and RNA pull-down assay [29]. RIP experiments were performed using a Magna RIP™ RNA-Binding Protein Immunoprecipitation Kit (Millipore, Temecula, CA, USA) according to the manufacturer’s instructions. The antibodies for RIP assays of JAK2 and STAT3 (ab108596 and ab76315) were from Abcam. For the RNA pull-down assay, breast cancer cells were quantitated and treated with 1 ml of cell lysis buffer for 72 h. Then they were rotated at 4 °C overnight after adding 1.5 μL RNase inhibitor, 10 μL streptavidin agarose beads and 500 pM biotin-labeled linc00514 oligos. Total RNAs were subjected to qRT-PCR analysis.

### Statistical analysis

All data were presented as mean ± standard deviation (SD). SPSS 18.0 (IBM, Armonk, NY, USA) and Graphpad Prism 5.0 was used to analyze data. The difference between two groups were compared by using the Student’s t-test. The difference among multiple groups was compared by using the one-way analysis of variance (ANOVA) followed by the LSD post hoc test. The difference of clinical characteristics was compared by using Chi-square test. Differences were considered statistically significant if *P* < 0.05.

## Results

### Highly expressed linc00514 promotes breast cancer malignancy

**Fig. 1:**
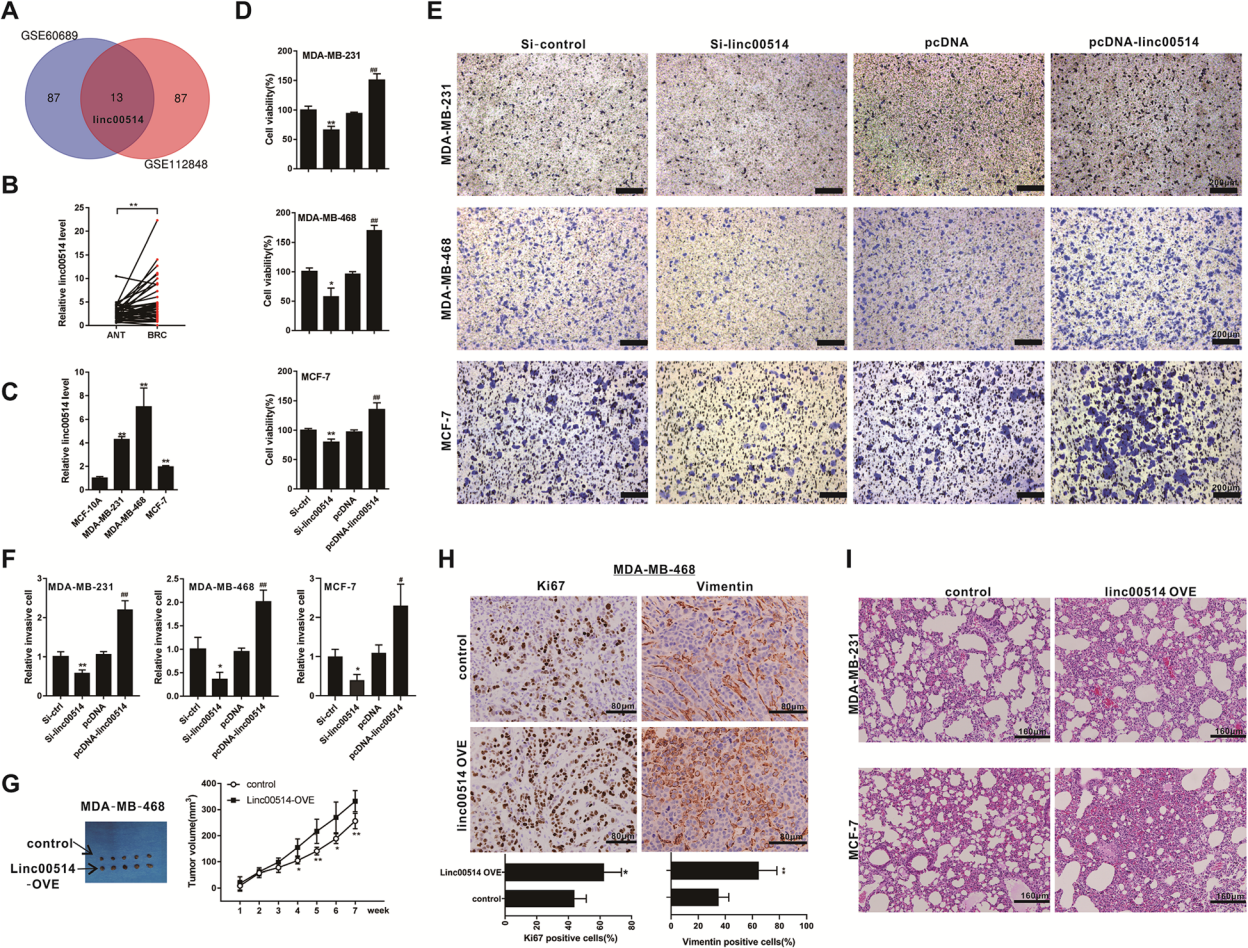
Highly expressed linc00514 promotes breast cancer malignancy. **a**. Top 100 dysregulated lncRNAs whose *P* < 0.05, log_2_FC > 2, or logFC < 0.5 were compared in the GSE60689 dataset and the GSE112848 dataset from the GEO database. **b**. The relative expression of linc00514 in tumor tissues (BRC) and in paracancer tissues (ANT) from 46 breast cancer patients was detected using qRT-PCR. **c**. The relative expression of linc00514 in human breast cancer cell lines (MDA-MB-231, MDA-MB-468, and MCF-7) and in the normal breast epithelial cell line (MCF-10A) was detected using qRT-PCR. **P < 0.01 vs MCF-10A. **d-f**. Human breast cancer cell lines (MDA-MB-231, MDA-MB-468, and MCF-7) were transiently transfected with linc00514 siRNAs (Si-linc00514) or linc00514 plasmids (pcDNA-linc00514) for 48 h. Cell viability was detected using MTT assay (**d**). Cell invasion was detected using Transwell assay (**e**, **f**). Scale bar = 200 μm. **g-h**. Female Balb/c nude mice were subcutaneously injected with the MDA-MB-468 cells (5 × 10^6^ cells) which were stably transfected with linc00514-overexpressing plasmids (linc00514-OVE) or the control plasmids (*n* = 5 in each group). The tumor volumes were detected every week (**g**). After 7 weeks, the Ki67 expression and the Vimentin expression in tumor tissues were detected using immunohistochemistry (**h**). Scale bar = 80 μm. **i**. Female Balb/c nude mice were intravenously injected with the MDA-MB-231/MCF-7 cells (1.5 × 10^6^ cells) which were stably transfected with linc00514-OVE (*n* = 5 in each group). The pulmonary metastatic tumor tissues were observed using HE staining. Scale Bar = 160 μm. Three independent experiments. *P < 0.05, **P < 0.01 vs control (Si-ctrl). ##*P* < 0.01 vs control (pcDNA)

In the GEO database (http://www.ncbi.nlm.nih.gov/geo/), we used ‘breast cancer’ as a key word to screen out the data of dysregulated lncRNAs in breast cancer tissues. From the GSE60689 dataset and the GSE112848 dataset, top 100 dysregulated lncRNAs whose *P* < 0.05, log_2_FC > 2, or logFC < 0.5 were compared. There are 13 lncRNAs in both datasets, including linc00514 (Fig. 1a). Among the 13 lncRNAs, we found that 9 of them were aberrantly expressed in clinical breast cancer tissues, and that linc00514, linc00964, and Clorf81 had the correlation with the clinical characteristics of the breast cancer patients (Fig. 1a & S1A). Since the fold change of linc00514 was more significant compared with linc00964 and Clorf81, we focused on linc00514 in the follow-up experiments. Linc00514 is located in chr16: 2,988,256-3,002,016, and has three major long non-coding transcripts of 313 bp, 3385 bp and 2216 bp in length (Fig. S1B). The PCR revealed that the 3385-nt Linc00514 (ENST00000571152) is a predominant and stable transcript in breast cancer cell lines and tissues [30] (Fig. S1C). The expression of linc00514 in tumor tissues and cells was compared. The relative expression of linc00514 in tumor tissues from 46 breast cancer patients was higher than that in paracancer tissues (*P* < 0.01, Fig. 1b). Similarly, the relative expression of linc00514 in human breast cancer cell lines (MDA-MB-231, MDA-MB-468, and MCF-7) was higher than that in the normal breast epithelial cell line (MCF-10A) (all *P* < 0.01, Fig. 1c). According to the relative linc00514 expression level of 46 patients, we divided them into the high linc00514 expression group (*n* = 23) and the low linc00514 expression group (*n* = 23). The higher level of linc00514 was related to the larger tumor size of breast cancer (*P* < 0.05, Table 1). These data indicated that the dysregulated linc00514 was correlated with the pathogenesis of breast cancer.

Then, the gain-of-function and the loss-of-function experiments were performed. In human breast cancer cell lines (MDA-MB-231, MDA-MB-468, and MCF-7), the siRNA-mediated linc00514 knockdown significantly inhibited the viability of breast cancer cells (*P* < 0.01), while the plasmid-mediated linc00514 overexpression significantly promoted the viability of breast cancer cells (*P* < 0.01, Fig. 1d). Similarly, the linc00514 knockdown markedly inhibited the invasion of breast cancer cells (*P* < 0.01), and the linc00514 overexpression markedly promoted the invasion of breast cancer cells (*P* < 0.01, Fig. 1e & f). In addition, the in vivo experiments indicated that the subcutaneous injection of the MDA-MB-468 cells which were stably transfected with linc00514-overexpressing plasmids (linc00514-OVE) increased the tumor volume of the mouse xenograft (Fig. 1g). The tumor tissues showed a higher Ki67 and Vimentin intensity in the linc00514-OVE group than that in the control group, indicating a more aggressive ability of the linc00514-overexpressing MDA-MB-468 cells (Fig. 1h). Meanwhile, the venous injection of the MDA-MB-231/MCF-7 cells which were stably transfected with linc00514-OVE promoted the pulmonary metastasis of the nude mice (Fig. 1i). Both in vitro and in vivo experiments suggested that the overexpression of linc00514 increased the malignancy of breast cancer.

### Overexpression of linc00514 in breast cancer cells promotes M2 polarization of macrophages

**Fig. 2:**
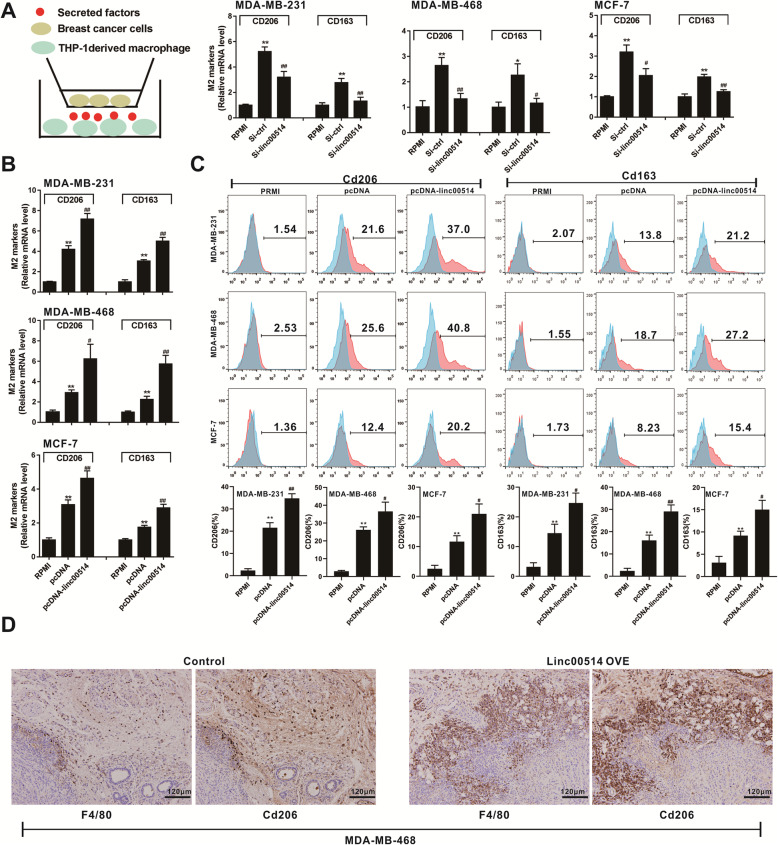
Overexpression of linc00514 in breast cancer cells promotes M2 polarization of macrophages. **a-b**. Human breast cancer cell lines (MDA-MB-231, MDA-MB-468, and MCF-7) were transiently transfected with linc00514 siRNAs (Si-linc00514) or linc00514 plasmids (pcDNA-linc00514) for 48 h before the co-culture. After co-culturing breast cancer cells with PMA-induced human monocyte THP-1 cells for 48 h using Transwell assay, we detected the relative mRNA levels of CD206 and CD163 in THP-1 derived macrophages, both of which were M2 polarization markers of macrophages. **c**. The percentage of CD206+ or CD163+ cells after overexpressing linc00514 was detected using flow cytometry. **d**. Female Balb/c nude mice were subcutaneously injected with the MDA-MB-468 cells (5 × 10^6^ cells) which were stably transfected with linc00514-overexpressing plasmids (linc00514-OVE) or the control plasmids (*n* = 5 in each group). After 7 weeks, the expression of F4/80 and CD206 in tumor tissues was detected using immunohistochemistry. Scale bar = 120 μm. Three independent experiments. ** *P* < 0.01 vs control (RPMI). ## *P* < 0.01 vs control (Si-ctrl or pcDNA)

After co-culturing human breast cancer cell lines (MDA-MB-231, MDA-MB-468, and MCF-7) with PMA-induced human monocyte THP-1 cells for 48 h, we found that the siRNA-mediated linc00514 knockdown in breast cancer cells reduced the relative mRNA levels of CD206 and CD163 in THP-1 derived macrophages, both of which were M2 polarization markers of macrophages (Fig. 2a), and that the pcDNA-mediated linc00514 overexpression in breast cancer cells increased the relative mRNA levels of CD206 and CD163 in THP-1 derived macrophages (Fig. 2b). In addition, the flow cytometry results showed an enhancement of the percentage of CD206+ or CD163+ cells after overexpressing linc00514 (Fig. 2c). Meanwhile, the overexpression of linc00514 in breast cancer cells inhibited the THP-1 mRNA levels of TNF-α, NOS2, and IL-6, which are M1 polarization markers of macrophages, while it promoted the expression of Arg-1 at both mRNA and protein levels, which is a M2 polarization marker (Fig. S2A & S2B). The in vivo experiments showed that the F4/80+ or CD206+ cells in tumor tissues from mouse xenograft were increased after the subcutaneous injection of the MDA-MB-468 cells which were stably transfected with linc00514-OVE (Fig. 2d). In addition, in linc00514-OVE tumor tissues, the Arg-1 expression (Fig. S2C) and the F4/80+ cells (Fig. S2D-i) were increased, and the percentages of iNOS+/TNF-α + cells in total F4/80+ cells were reduced (Fig. S2D-ii & 2E). Furthermore, the interference of STAT3 (the transfection of si-STAT3) in linc00514-OVE breast cancer cells elevated the mRNA levels of TNF-α, NOS2, and IL-6 (Fig. S2F), and reduced the expression of Arg-1 at both mRNA and protein levels (Fig. S2G). These data indicated that overexpressing linc00514 in breast cancer cells promotes M2 polarization of macrophages.

### pSTAT3 and Jagged1 participates in the breast cancer metastasis and M2 polarization of macrophages

**Fig. 3:**
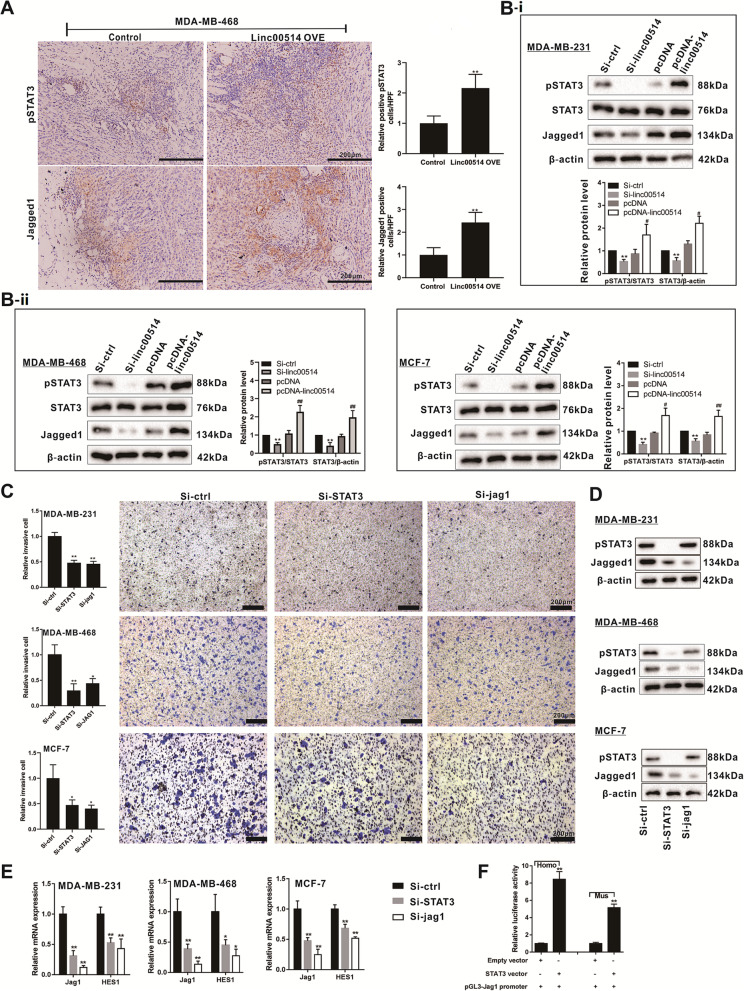
Linc00514 positively regulates the protein expression of pSTAT3 and Jagged1. **a**. Female Balb/c nude mice were subcutaneously injected with the MDA-MB-468 cells (5 × 10^6^ cells) which were stably transfected with linc00514-overexpressing plasmids (linc00514-OVE) or the control plasmids (*n* = 5 in each group). After 7 weeks, the expression of pSTAT3 and Jagged1 in tumor tissues was detected using immunohistochemistry. Scale bar = 200 μm. **b**. Human breast cancer cell lines (MDA-MB-231, MDA-MB-468, and MCF-7) were transiently transfected with linc00514 siRNAs (Si-linc00514) or linc00514 plasmids (pcDNA-linc00514) for 48 h. The expression of pSTAT3, STAT3, and Jagged1 was detected using western blot analysis. **c-e**. Human breast cancer cell lines (MDA-MB-231, MDA-MB-468, and MCF-7) were transiently transfected with STAT3 siRNAs (Si-STAT3) or JAG1 siRNAs (Si-JAG1) for 48 h. Cell invasion was detected using MTT assay (c). Scale bar = 200 μm. The protein level of pSTAT3 and Jagged1 was detected using western blot analysis (**d**). The relative mRNA expression of JAG1 and HES1 was detected using qRT-PCR (**e**). **f**. The 293 T cells were transfected with human or mouse STAT3 vectors or empty vectors as well as JAG1 promoter plasmids. The relative luciferase activity was detected using luciferase reporter assay. Three independent experiments. *P < 0.05, ** *P* < 0.01 vs control (Si-ctrl). #*P* < 0.05, ##*P* < 0.01 vs control (Si-ctrl or pcDNA)

To elucidate the mechanism of linc00514 in promoting breast cancer malignancy, we detected the expression of pSTAT3 and Jagged1 in mouse tumor tissues and breast cancer cell lines after overexpressing linc00514. The in vivo experiments showed that the expression of pSTAT3 and Jagged1 in tumor tissues from the mouse xenograft was increased after the subcutaneous injection of the MDA-MB-468 cells which were stably transfected with linc00514-OVE (Fig. 3a). The in vitro experiments showed that the siRNA-mediated linc00514 knockdown in breast cancer cells reduced the protein level of pSTAT3 and Jagged1, and that the pcDNA-mediated linc00514 overexpression in breast cancer cells increased the protein level of pSTAT3 and Jagged1 (Fig. 3b-i & b-ii). The siRNA-mediated STAT3 knockdown or JAG1 knockdown markedly inhibited the invasion of breast cancer cells (Fig. 3c). Meanwhile, the knockdown of STAT3 reduced the Jagged1 protein level, while the knockdown of JAG1 did not affect the STAT3 protein level (Fig. 3d), suggesting that STAT3 may be the upstream regulator of Jagged1. STAT3 knockdown or JAG1 knockdown could reduce the expression of HES1, which is a downstream molecule of the Notch signaling pathway (Fig. 3e). In addition, the luciferase reporter assay showed that the STAT3 vector could increase the luciferase activity in 293 T cells which were transfected with human or mouse JAG1 promoter plasmids (Fig. 3f), indicating that STAT3 activates JAG1 transcription. These data suggested that STAT3 promotes Jagged1 expression partly via increasing JAG1 transcription, thus subsequently increasing HES1 expression.

**Fig. 4:**
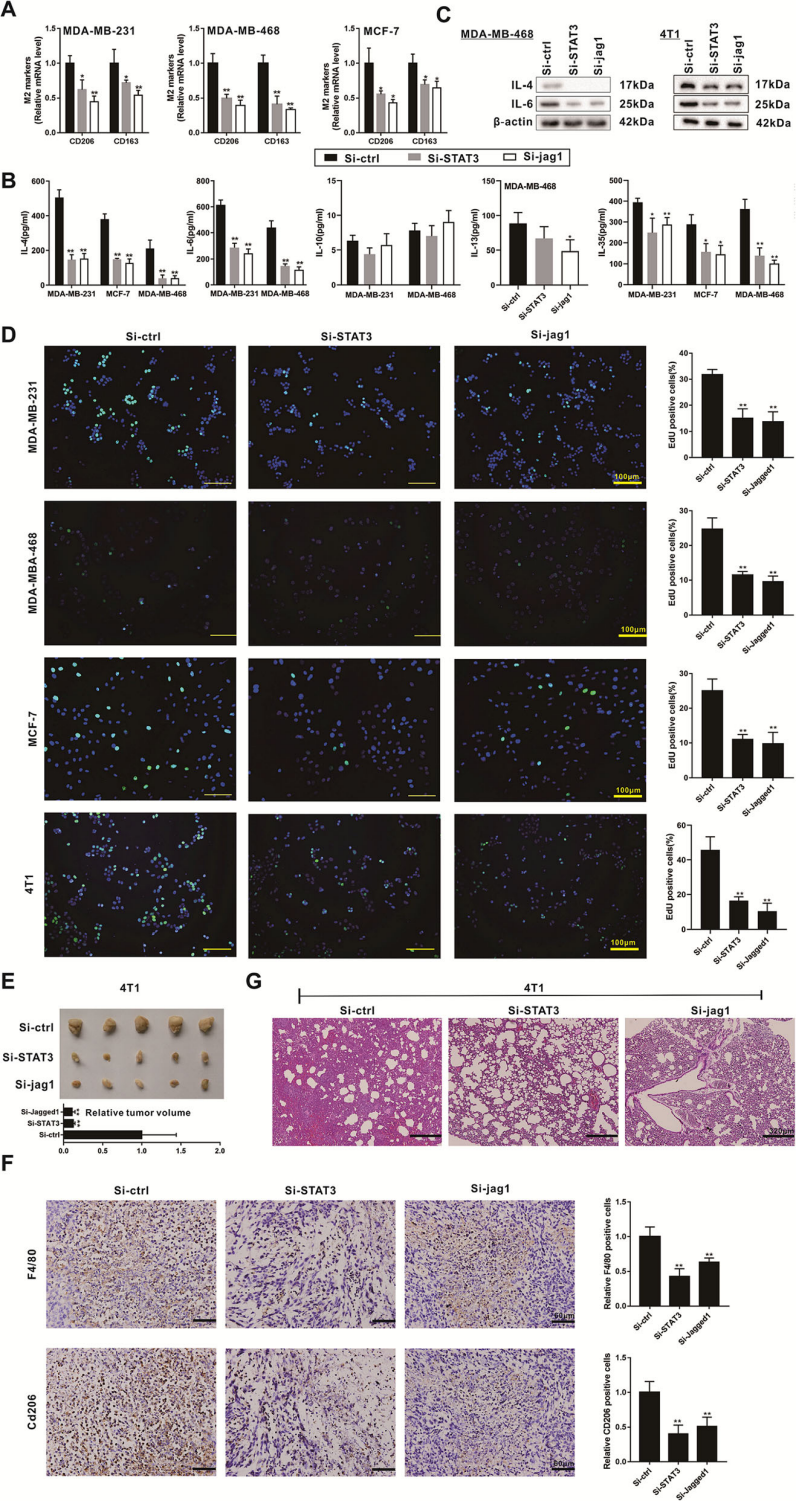
STAT3 and Jagged1 participates in the breast cancer metastasis and M2 polarization of macrophages. **a**. Human breast cancer cell lines (MDA-MB-231, MDA-MB-468, and MCF-7) were transiently transfected with STAT3 siRNAs (Si-STAT3) or JAG1 siRNAs (Si-JAG1) for 48 h before the co-culture. After co-culturing breast cancer cells with PMA-induced human monocyte THP-1 cells for 48 h using Transwell assay, we detected the relative mRNA levels of CD206 and CD163 in THP-1 derived macrophages, both of which were M2 polarization markers of macrophages. **b-d**. Human breast cancer cell lines (MDA-MB-231, MDA-MB-468, and MCF-7) and mouse breast cancer cell line (4 T1) were transiently transfected with STAT3 siRNAs (Si-STAT3) or JAG1 siRNAs (Si-JAG1) for 48 h. The level of IL-4, IL-6, IL-10, IL-13, and IL-35 in supernatants was detected using ELISA (**b**). The protein level of IL-4 and IL-6 in breast cells was detected using western blot analysis (**c**). The cell proliferation was detected using EdU labeling detection (**d**). Scale Bar =100 μm. **e-f**. Female Balb/c mice were subcutaneously injected with the mouse breast cancer cell line (4 T1, 5 × 10^5^ cells) which were transfected with STAT3 siRNAs (Si-STAT3), JAG1 siRNAs (Si-JAG1), or the control siRNAs (Si-ctrl) (*n* = 10 in each group). The tumor tissues were collected and measured on the 20th day (**e**). The F4/80 and CD206 expressions in tumor tissues were detected using immunohistochemistry (**f**). Scale bar = 60 μm. **g**. Female Balb/c mice were intravenously injected with the 4 T1 cells (1.5 × 10^5^ cells) which were transfected with STAT3 siRNAs (Si-STAT3), JAG1 siRNAs (Si-JAG1), or the control siRNAs (Si-ctrl) (*n* = 5 in each group). The pulmonary metastatic tumor tissues were observed using HE staining. Scale Bar = 320 μm. Three independent experiments. *P < 0.05, **P < 0.01 vs control (Si-ctrl)

After co-culturing human breast cancer cell lines (MDA-MB-231, MDA-MB-468, and MCF-7) with PMA-induced human monocyte THP-1 cells for 48 h, we found that either STAT3 knockdown or JAG1 knockdown in breast cancer cells reduced the relative mRNA levels of CD206 and CD163 in THP-1 derived macrophages (Fig. 4a). In addition, STAT3 knockdown or JAG1 knockdown reduced the secretion and protein expression of IL-4 and IL-6 in breast cancer cells (Fig. 4b & c), it also inhibited the proliferation of breast cancer cells (Fig. 4d). We also detected the effect of STAT3 knockdown or JAG1 knockdown on the secretions of IL-10, IL-13, and IL-35, which have been reported to participate in M2 polarization of macrophages [31, 32], and found that STAT3 knockdown or JAG1 knockdown markedly reduced the IL-35 secretion (Fig. 4b).

The in vivo experiments showed that the tumor volumes of mouse primary breast cancer were obviously smaller after the subcutaneous injection of the 4 T1 cells which were transfected with STAT3 siRNAs (Si-STAT3) or JAG1 siRNAs (Si-JAG1) (Fig. 4e). The death rates of mouse in the Si-ctrl group, the Si-STAT3 group, and the Si-JAG1 group were 50% (5/10), 20% (2/10), and 30% (3/10), respectively, and the survival curve was shown in Fig. S3A. After STAT3 knockdown or JAG1 knockdown, the protein expression of Arg-1 in tumor tissues was reduced (Fig. S3B). The immunochemistry results showed a decreased expression of F4/80 and CD206 in the Si-STAT3 group and the Si-JAG1 group (Fig. 4f & S3C), and the percentages of iNOS+/TNF-α + cells in total F4/80+ cells were increased (Fig. S3C & S3D). Meanwhile, the venous injection of the 4 T1 cells which were transfected with Si-STAT3 or Si-JAG1 inhibited the pulmonary metastasis of the nude mice (Fig. 4g).

### Overexpression of linc00514 promotes proliferation and invasion of breast cancer cells and M2 polarization of macrophages via regulating STAT3

**Fig. 5:**
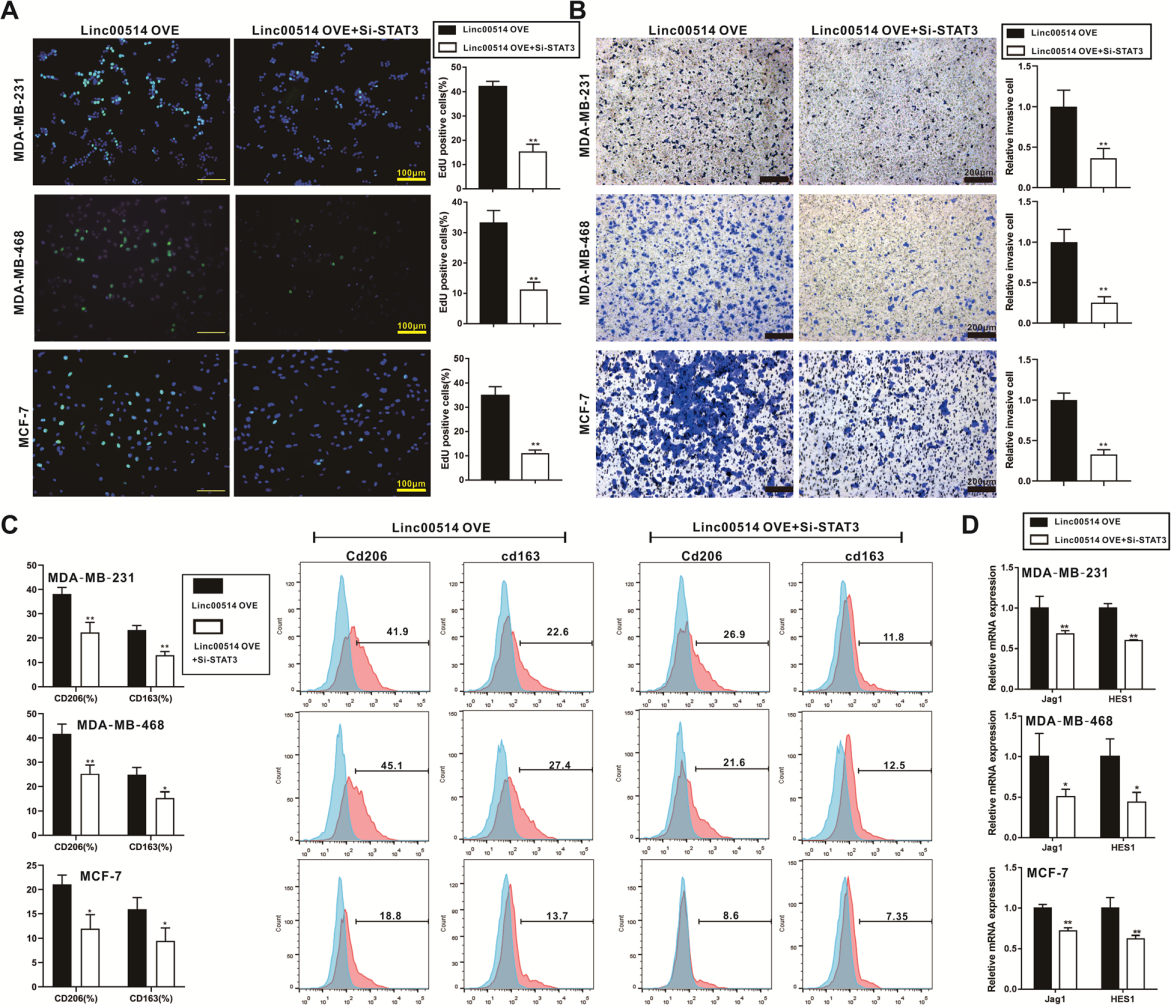
Overexpression of linc00514 promotes proliferation and invasion of breast cancer cells and M2 polarization of macrophages via regulating STAT3. Human breast cancer cell lines (MDA-MB-231, MDA-MB-468, and MCF-7) which were stably transfected with linc00514 plasmids (linc00514-OVE) were then transfected with STAT3 siRNAs (Si-STAT3) for 48 h. **a**. The cell proliferation was detected using EdU labeling detection. Scale Bar =100 μm. **b**. The cell invasion was detected using Transwell assay. Scale bar = 200 μm. **c-d**. After co-culturing breast cancer cells with PMA-induced human monocyte THP-1 cells for 48 h using Transwell assay, we detected the relative percentage of CD206+ and CD163+ in THP-1 derived macrophages using flow cytometry (**c**). The relative mRNA expression of JAG1 and HES1 was detected using qRT-PCR (**d**). Three independent experiments. *P < 0.05, **P < 0.01 vs control (linc00514-OVE)

Human breast cancer cell lines (MDA-MB-231, MDA-MB-468, and MCF-7) which were stably transfected with linc00514 plasmids (linc00514-OVE) were then transfected with STAT3 siRNAs (Si-STAT3) for 48 h. The Si-STAT3 transfection reduced the proliferation (Fig. 5a) and the invasion (Fig. 5b) of linc00514-OVE breast cancer cells. The Si-STAT3 transfection also reduced the percentage of CD206+ or CD163+ THP-1 derived macrophages after the co-culture with linc00514-OVE breast cancer cells (Fig. 5c). In addition, the Si-STAT3 transfection reduced the relative mRNA expression of JAG1 and HES1 in linc00514-OVE breast cancer cells (Fig. 5d). Our findings indicated that overexpression of linc00514 promotes proliferation and invasion of breast cancer cells and M2 polarization of macrophages via reducing STAT3 expression.

### Linc00514 increases phosphorylation of STAT3

**Fig. 6:**
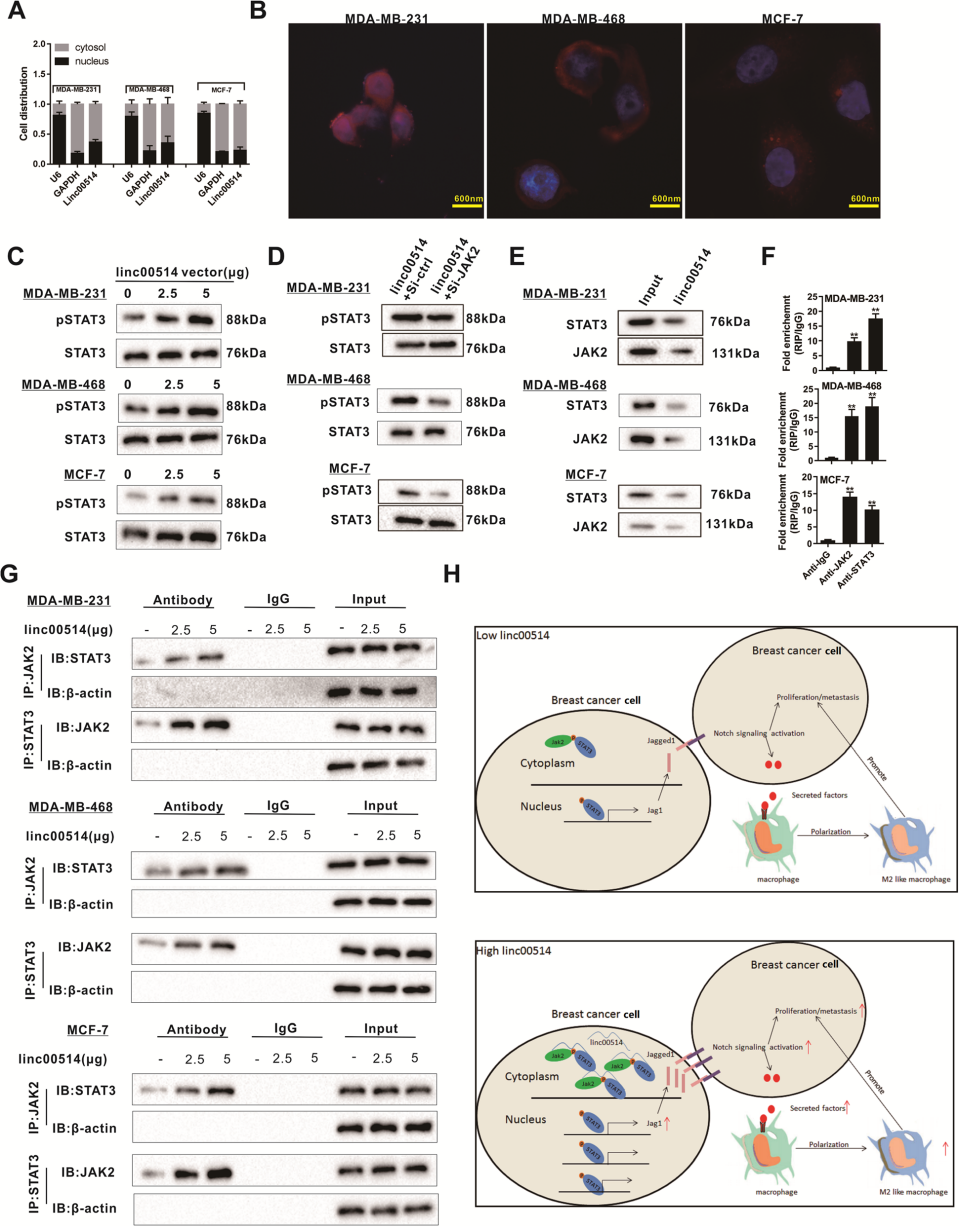
Linc00514 increases phosphorylation of STAT3. **a-b**. The distribution and localization of linc00514 in human breast cancer cell lines (MDA-MB-231, MDA-MB-468, and MCF-7) were detected using cytosol/nucleus qRT-PCR (**a**) and FISH (**b**). **c**. Human breast cancer cell lines (MDA-MB-231 and MCF-7) were transfected with the increasing concentration of linc00514 vectors (from 0 to 5 μg). The protein level of pSTAT3 and STAT3 was detected using western blot analysis. **d**. The siRNA-mediated JAK2 knockdown reduced the pSTAT3 protein level which was increased by linc00514 overexpression. **e-f**. The RNA pull-down experiment (**e**) and the RIP experiment (**f**) confirmed that linc00514 could bind with STAT3 and JAK2. **g**. Human breast cancer cell lines (MDA-MB-231 and MCF-7) were transfected with the increasing concentration of linc00514 vectors (from 0 to 5 μg). The levels of STAT3 and JAK2 pulled down by linc00514 probes were detected using the RNA pull-down experiment. **h**. Schema depicting the mechanisms of linc00514 in breast cancer growth and metastasis. Three independent experiments. **P < 0.01 vs control (Anti-IgG)

In human breast cancer cell lines (MDA-MB-231, MDA-MB-468, and MCF-7), linc00514 was mainly distributed in cytoplasm (Fig. 6a). FISH results also confirmed the cytosol localization of linc00514 (red fluorescence) in breast cancer cells (Fig. 6b). The gradual enhancement of the linc00514 vectors (from 0 to 5 μg) during cell transfection increased the protein level of pSTAT3, while it did not affect the protein level of STAT3 (Fig. 6c), suggesting linc00514 could increase the phosphorylation of STAT3. JAK2 is a well-defined phosphokinase and is involved in the phosphorylation of STAT3. In this study, the siRNA-mediated JAK2 knockdown reduced the pSTAT3 protein level which was increased by linc00514 overexpression (Fig. 6d). The RNA pull-down experiment (Fig. 6e) and the RIP experiment (Fig. 6f) confirmed that linc00514 could bind with STAT3 and JAK2. In addition, the gradual enhancement of the linc00514 vectors (from 0 to 5 μg) during cell transfection promoted the binding between JAK2 and STAT3 (Fig. 6g). Taken together, our findings indicated that linc00514 increased the phosphorylation of STAT3 partly through the recruitment of JAK2 and STAT3.

## Discussion

Although the interaction between TAMs and tumor cells in TME has been established, the mechanisms underlying the modulation of TAM polarization are still limited. In the current study, we found that the highly expressed linc00514 is associated with breast cancer tumorigenicity and macrophage M2 polarization. Mechanistically, overexpression of linc00514 stimulated IL-6 and IL-4 secretion of breast cancer cells via STAT3/Jagged1 axis, and promoted monocyte polarization into M2-like macrophages and increase the tumor progression (Fig. 6h). Our findings revealed a novel lncRNA, linc00514, which exerts the oncogenic function in breast cancer.

As reported, various lncRNAs have been reported to participate in the progression of breast cancer. For example, lncRNA APOC1P1–3 has been found to inhibit breast cancer cell apoptosis by decreasing α-tubulin acetylation [13]; lncRNA NORAD has been reported to suppress breast cancer metastasis by sequestering S100P [15]. Inspired by these studies, we used the GEO database and screened some dysregulated lncRNAs. Among them, linc00514 was dysregulated in both GSE60689 and GSE112848 profiles. Linc00514 is located at the Chromosome 16p13.3 with 3221 bp in length, and it has been shown to play a cancer-promoting role in papillary thyroid cancer [30]. Consistent with the study, we found that the expression of linc00514 was significantly higher in both clinical breast cancer tissues and breast cancer cells. After downregulating linc00514 expression by using RNA interfering technology (RNAi) in breast cancer cells, the invasion and proliferation were significantly reduced, and the M2 polarization of macrophages was suppressed. Such responses were negated after upregulating linc00514 by using linc00514 plasmids in breast cancer cells. In addition, the in vivo experiments showed that the linc00514-overexpressing breast cancer cells have a progressive ability of growth and metastasis. These data indicated that linc00514 acts as an oncogene in breast cancer cells, and the therapeutic strategy targeting linc00514 may provide good effects, although more researches are still needed.

STAT3 belongs to the STAT transcription factor family. It is phosphorylated by phosphokinases such as JAK2 and enters the nucleus to modulate the gene expression [33]. As a transcription factor, STAT3 has been reported to promote the expression of cancer-promoting molecules such as Bcl-2, Survivin, and c-Myc [34]. The high level of STAT3 and pSTAT3 is closely related to the tumor progression and poor prognosis of cancer patients [35]. In breast cancer, Wang et al. [36] found that the JAK/STAT3 is critical for breast cancer stem cell self-renewal and chemoresistance by regulating lipid metabolic genes. Chang et al. [37] found that JAK2/STAT3 mediated breast cancer cell metastasis and proliferation. In addition, a study conducted by Wang et al. [20] indicated that cytosol STAT3 could be directly bound by lncRNA lnc-DC, therefore the phosphorylation of STAT3 was promoted on tyrosine-705, and preventing STAT3 binding to and being dephosphorylated by SHP1. Based on their study, we found that STAT3 could also be bound with linc00514 as STAT3 was detected in linc00514 pulled-down compounds and linc00514 expression was increased in anti-STAT3-immunoprecipitated compounds. Similarly, JAK2 was also proven to be bound with linc00514 using RIP and RNA pull-down assays. In addition, linc00514 was mainly distributed in cytoplasm of breast cancer cells. We believed that the cytosol linc00514 could recruit STAT3 to bind with JAK2, therefore increasing pSTAT3, promoting transcription activation of JAG1. However, whether the binding between linc00514 and JAK2/STAT3 affect the cell translocation of pSTAT3 deserve further investigations.

The Notch signaling pathway has been well-defined in cell development and differentiation. Recently, accumulating studies have revealed the aberrant activation of the Notch signaling pathway is involved in tumorigenesis, including breast cancer [38]. The activation of the Notch signaling pathway requires the interaction between ligands and receptors in adjacent cells. Jagged1 is one of the major ligands in the Notch signaling pathway, and when Jagged1 binds to a Notch receptor (Notch 1–4), the Notch intracellular domain (NICD) will be discharged within the cytoplasm and translocated to nucleus, subsequently transcriptional activating downstream target genes like HES1 [39]. In our study, the siRNA-mediated JAG1 knockdown or STAT3 knockdown significantly reduced HES1 expression, and STAT3 knockdown reduced JAG1 and HES1 expressions which were raised by linc00514 overexpression. Our findings revealed that linc00514 positively regulates Jagged1-mediated Notch signaling pathway through STAT3. On the other hand, Notch signaling pathway has been extensively implicated in determining cell fates of immune cells, including macrophages. The activation of Jagged1-mediated Notch signaling pathway affects the differentiation and polarization of macrophages [40, 41]. However, whether the polarization of macrophages can be regulated by the activation of Jagged1-mediated Notch signaling pathway in tumor cells is rarely reported. In the current study, we co-cultured breast cancer cells with monocytic THP-1 cells using Transwell assay, and we found that the co-culture increased M2-like macrophages. Meanwhile, we found that the siRNA-mediated JAG1 knockdown impairs the secretion and expression of IL-4 and IL-6 of breast cancer cells. We believed that the induction of M2 polarization of macrophages is related to the activation of Jagged1-mediated Notch signaling pathway and the increased secretions of IL-4 and IL-6 in breast cancer cells. Consistent with our findings, Lin et al. [12] found that the Notch signaling pathway in colon cancer cells can affect the polarization and recruitment of macrophages by secreting IL-4. As reported, apart from ligand binding, Notch expression is also regulated by inflammatory cytokines [42]. Whether inflammatory cytokines and other components in TME affects the polarization of macrophages will be further explored in our future researches.

In the current study, we investigated the effect of linc00514 on the breast cancer progression via two directions. One is that linc00514 regulated the growth and metastasis of breast cancer, and the other is that linc00514 regulated the polarization of macrophages. In addition, is has been reported that the polarization of macrophages contributes to the progression of tumor growth and metastasis [6, 43, 44]. Our findings also indicated that the increased M2 polarization of macrophages is related to the malignancy of breast cancer. However, the specific mechanisms underlying the relationship between M2 polarization of macrophages and the tumor growth remain unclear, and we will focus on them in our future investigations.

## Conclusions

In conclusion, our findings indicated that linc00514 exerts the cancer-promoting effects in breast cancer via modulating tumorigenicity and M2 polarization of TAMs. The mechanism is partly through activating Jagged1-mediated Notch signaling pathway via increasing pSTAT3. Our study has provided a novel target of treating breast cancer, although more evidence is still needed.

## Supplementary information

**Additional file 1: Figure S1.** Screening of the candidate lncRNAs. **A**. The expressions in tumor tissues (BRC) and paracancer tissues (ANT) and the clinical correlations of candidate lncRNAs. **B**. The location of Linc00514 in Chr16. **C**. The expression of Linc00514 in breast cancer cell lines and tissues was detected using PCR. *P < 0.05, **P < 0.01.

**Additional file 2: Figure S2.** The effect of linc00514 overexpression on the differentiation of macrophages. **A-B**. Human breast cancer cell lines (MDA-MB-231, MDA-MB-468, and MCF-7) were transiently transfected with linc00514 plasmids (pcDNA-linc00514) for 48 h before the co-culture. After co-culturing breast cancer cells with PMA-induced human monocyte THP-1 cells for 48 h using Transwell assay, we detected the relative mRNA levels of M1 polarization markers, including TNF-α, NOS2, and IL-6, and the mRNA and the protein levels of M2 polarization marker Arg-1 in THP-1 derived macrophages using qRT-PCR and western blot analysis, respectively. **C-E**. Female Balb/c nude mice were subcutaneously injected with the MDA-MB-468 cells (5 × 10^6^ cells) which were stably transfected with linc00514-overexpressing plasmids (linc00514-OVE) or the control plasmids (*n* = 5 in each group). After 7 weeks, the expression of Arg-1, the percentages of F4/80+ cells, and the percentage of iNOS+/TNF-α + cells in total F4/80+ cells in tumor tissues were detected using immunofluorescence staining. Scale bar = 100 μm. **F-G**. The interference of STAT3 (the transfection of si-STAT3) in linc00514-OVE breast cancer cells elevated the mRNA levels of TNF-α, NOS2, and IL-6, and reduced the expression of Arg-1 at both mRNA and protein levels. Three independent experiments. *P < 0.05, **P < 0.01 vs control (RPMI) or linc00514-OVE. #*P* < 0.05, ##*P* < 0.01 vs pcDNA.

**Additional file 3: Figure S3.** The effect of STAT3 knockdown or JAG1 knockdown on the differentiation of macrophages. Female Balb/c mice were subcutaneously injected with the mouse breast cancer cell line (4 T1, 5 × 10^5^ cells) which were transfected with STAT3 siRNAs (Si-STAT3), JAG1 siRNAs (Si-JAG1), or the control siRNAs (Si-ctrl) (*n* = 10 in each group). **A**. The survival curves. **B**. The protein expression of Arg-1 in tumor tissues was detected using western blot analysis. **C-D**. The percentages of F4/80+ cells, and the percentage of iNOS+/TNF-α + cells in total F4/80+ cells in tumor tissues were detected using immunofluorescence staining. Scale bar = 50 μm. Three independent experiments. *P < 0.05, **P < 0.01 vs control (Si-ctrl).

## Data Availability

The datasets used and/or analyzed during the current study are available from the corresponding author on reasonable request.

## Abbreviations

TAM: Tumor-associated macrophages
TME: Tumor microenvironment
IL-6: Interleukin-6
ncRNA: Non-coding RNA
lncRNA: Long non-coding RNA
qRT-PCR: Quantitative real-time PCR
RIP: RNA immunoprecipitation
FBS: Fetal bovine serum
STAT3: Signal transducer and activator of transcription 3
pSTAT3: Phosphorylated STAT3
MTT: 3-(4,5-Dimethylthiazol-2-yl)-2,5-diphenyltetrazolium bromide
EdU: 5-Ethynyl-2-deoxyuridine
siRNA: Small interfering RNA
HE: Hematoxylin-eosin
NC: Negative control
PBS: Phosphate buffer saline
ELISA: Enzyme-linked immunosorbent assay
RNAi: RNA interfering technology
NICD: Notch intracellular domain
