# Supplementary figures and images for: Linc00514 promotes breast cancer metastasis and M2 polarization of tumor-associated macrophages via Jagged1-mediated notch signaling pathway
Source: J Exp Clin Cancer Res. 2020 Sep 17;39:191. doi: 10.1186/s13046-020-01676-x (PMC7500027; doi:10.1186/s13046-020-01676-x)

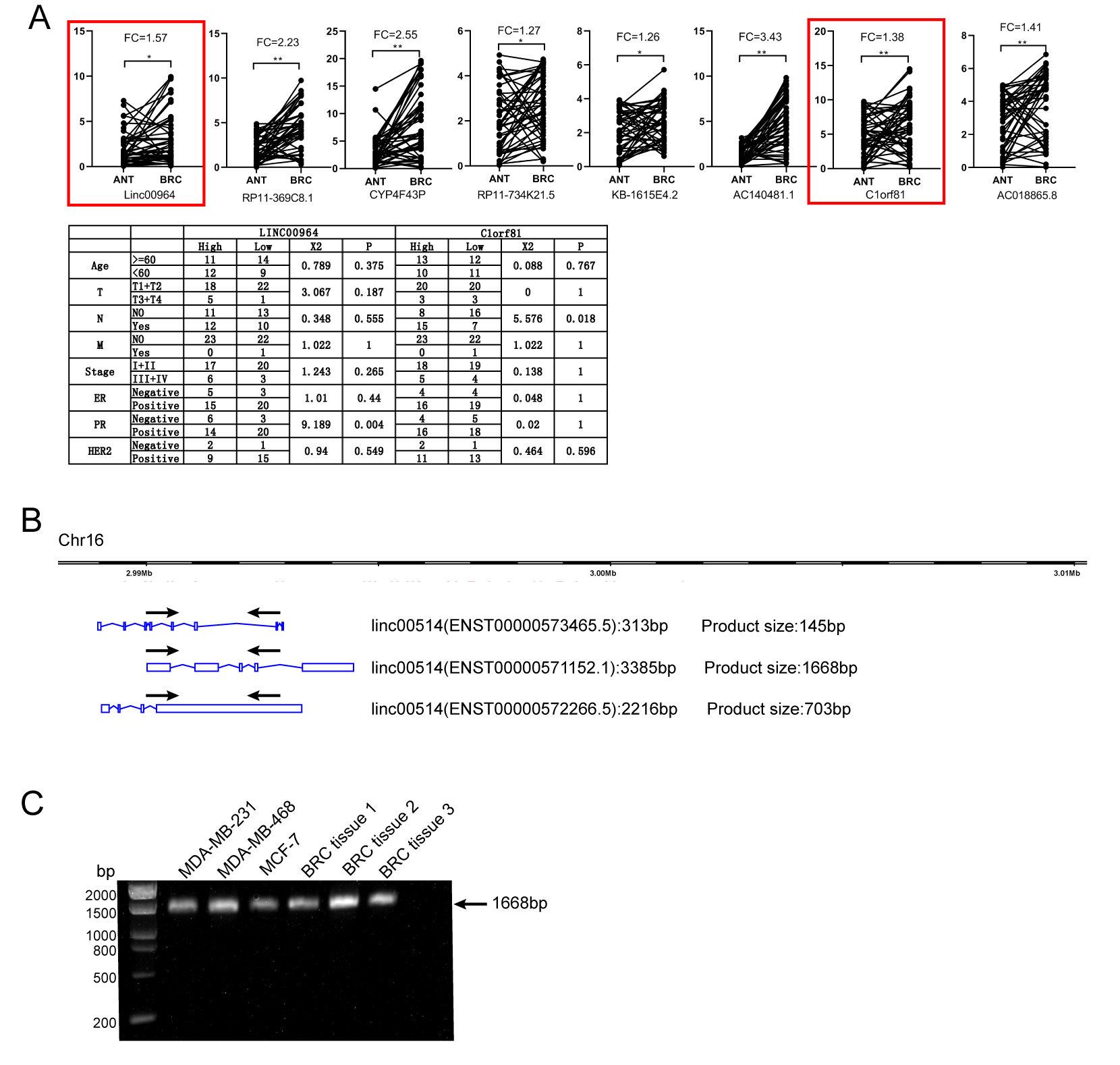

Supplement: Supplementary file 1 — Additional file 1: Figure S1. Screening of the candidate lncRNAs. A. The expressions in tumor tissues (BRC) and paracancer tissues (ANT) and the clinical correlations of candidate lncRNAs. B. The location of Linc00514 in Chr16. C. The expression of Linc00514 in breast cancer cell lines and tissues was detected using PCR. *P < 0.05, **P < 0.01. [file 13046_2020_1676_MOESM1_ESM.tif]

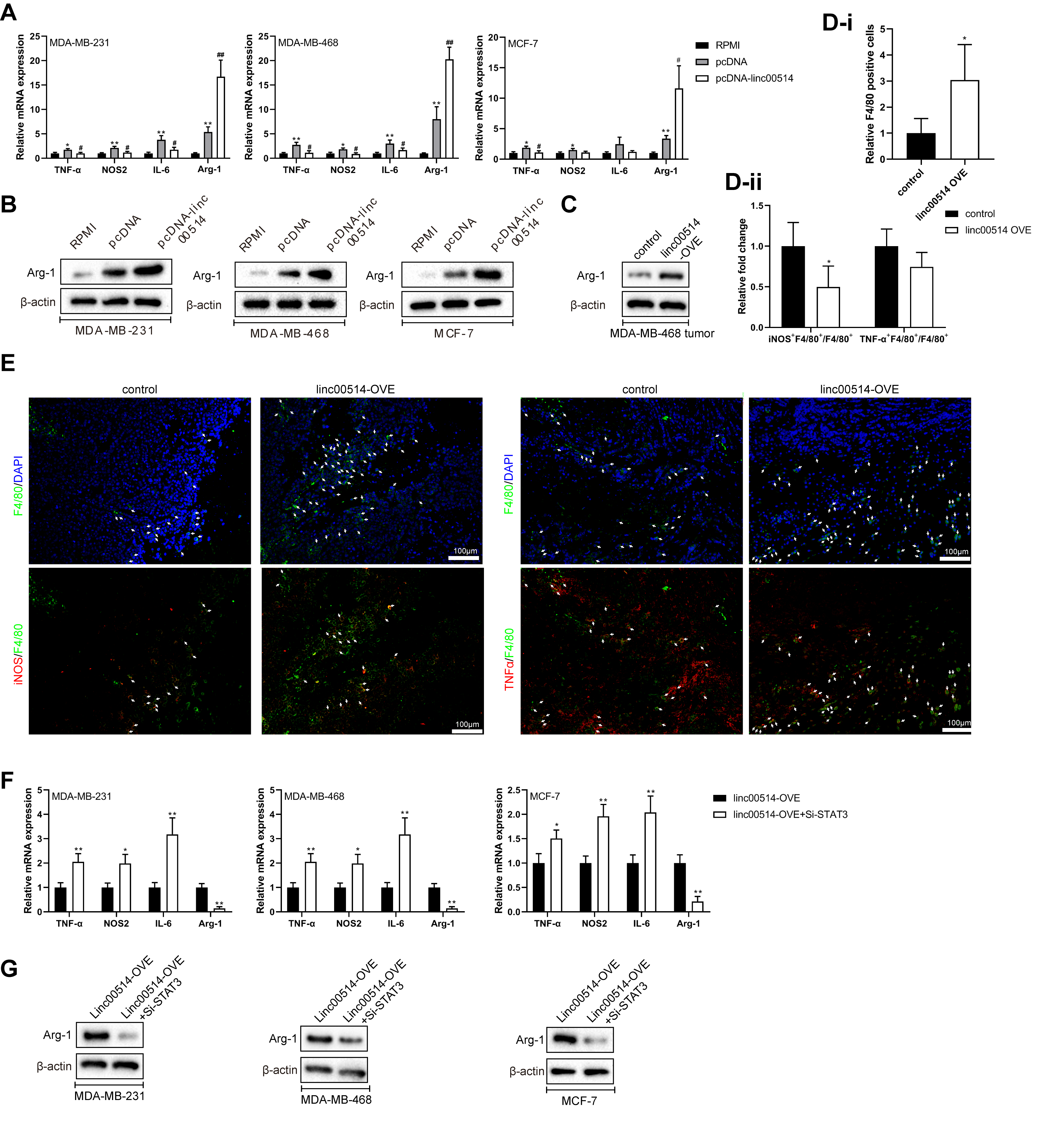

Supplement: Supplementary file 2 — Additional file 2: Figure S2. The effect of linc00514 overexpression on the differentiation of macrophages. A-B. Human breast cancer cell lines (MDA-MB-231, MDA-MB-468, and MCF-7) were transiently transfected with linc00514 plasmids (pcDNA-linc00514) for 48 h before the co-culture. After co-culturing breast cancer cells with PMA-induced human monocyte THP-1 cells for 48 h using Transwell assay, we detected the relative mRNA levels of M1 polarization markers, including TNF-α, NOS2, and IL-6, and the mRNA and the protein levels of M2 polarization marker Arg-1 in THP-1 derived macrophages using qRT-PCR and western blot analysis, respectively. C-E. Female Balb/c nude mice were subcutaneously injected with the MDA-MB-468 cells (5 × 10^6^ cells) which were stably transfected with linc00514-overexpressing plasmids (linc00514-OVE) or the control plasmids (n = 5 in each group). After 7 weeks, the expression of Arg-1, the percentages of F4/80+ cells, and the percentage of iNOS+/TNF-α + cells in total F4/80+ cells in tumor tissues were detected using immunofluorescence staining. Scale bar = 100 μm. F-G. The interference of STAT3 (the transfection of si-STAT3) in linc00514-OVE breast cancer cells elevated the mRNA levels of TNF-α, NOS2, and IL-6, and reduced the expression of Arg-1 at both mRNA and protein levels. Three independent experiments. *P < 0.05, **P < 0.01 vs control (RPMI) or linc00514-OVE. #P < 0.05, ##P < 0.01 vs pcDNA. [file 13046_2020_1676_MOESM2_ESM.tif]

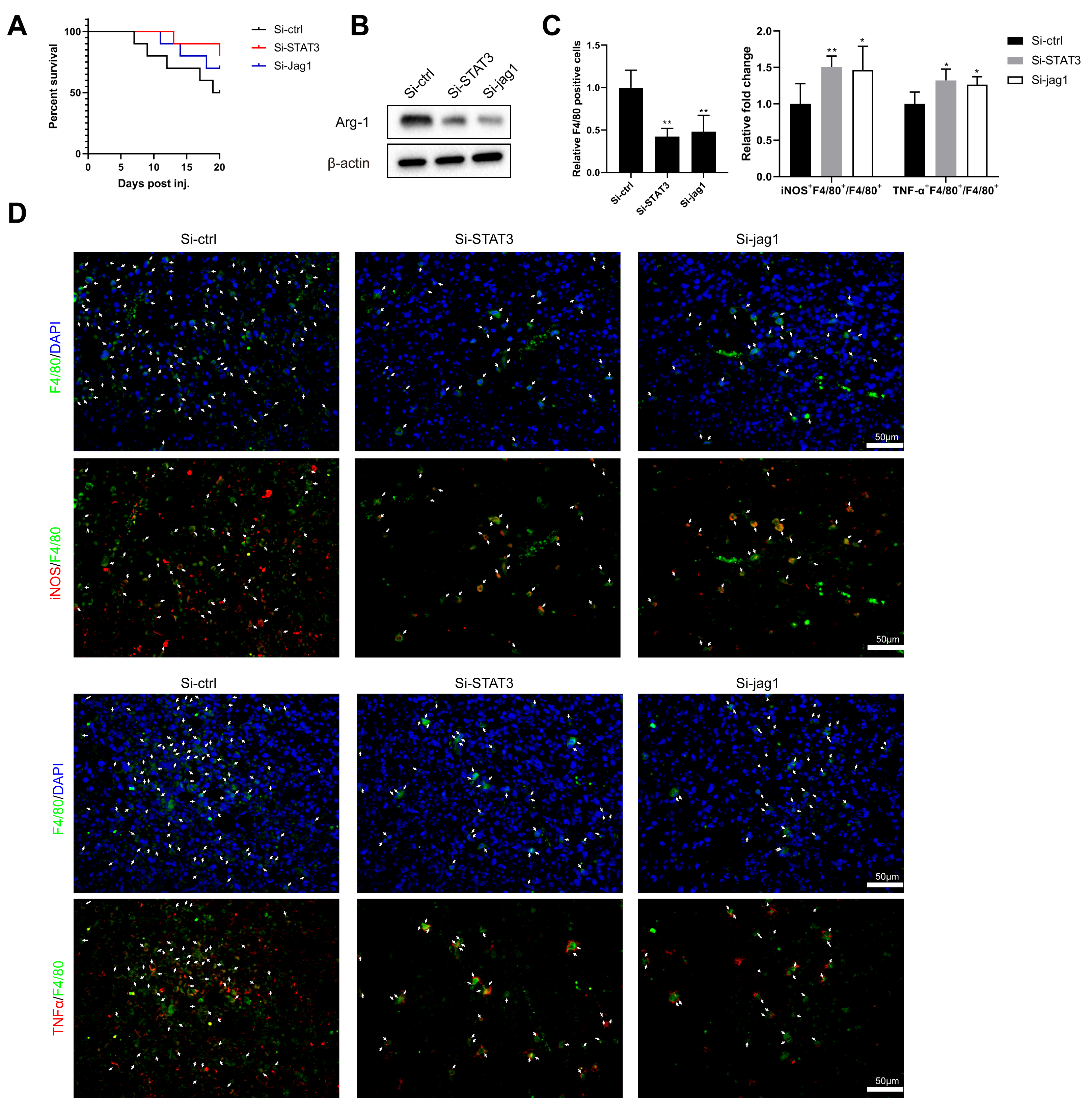

Supplement: Supplementary file 3 — Additional file 3: Figure S3. The effect of STAT3 knockdown or JAG1 knockdown on the differentiation of macrophages. Female Balb/c mice were subcutaneously injected with the mouse breast cancer cell line (4 T1, 5 × 10^5^ cells) which were transfected with STAT3 siRNAs (Si-STAT3), JAG1 siRNAs (Si-JAG1), or the control siRNAs (Si-ctrl) (n = 10 in each group). A. The survival curves. B. The protein expression of Arg-1 in tumor tissues was detected using western blot analysis. C-D. The percentages of F4/80+ cells, and the percentage of iNOS+/TNF-α + cells in total F4/80+ cells in tumor tissues were detected using immunofluorescence staining. Scale bar = 50 μm. Three independent experiments. *P < 0.05, **P < 0.01 vs control (Si-ctrl). [file 13046_2020_1676_MOESM3_ESM.tif]
